# Return to Play After a Lateral Ligament Ankle Sprain

**DOI:** 10.1007/s12178-020-09631-1

**Published:** 2020-05-06

**Authors:** P. D’Hooghe, F. Cruz, K. Alkhelaifi

**Affiliations:** Department of Orthopaedic Surgery, Aspetar Sports Medicine and Orthopedic Surgery Hospital, Sports City Street 1, Aspire Zone, P.O. Box: 29222, Doha, Qatar

**Keywords:** Ankle injuries, Ligament injuries, Rehabilitation, Return to play

## Abstract

**Purpose of Review:**

The purpose of this review is to describe the current evidence on the most common sports-related ankle injuries. Joint anatomy, epidemiology, clinical findings, diagnostic approach, and treatment are presented with a specific focus on the available evidence towards return to play.

**Recent Findings:**

Recent findings show that ankle sprain is the most common injury in the world of sports. Bony fractures, cartilage defects, and syndesmotic lesions are frequently seen in association with the more severe type of ankle sprains.

**Summary:**

In summary, the majority of the athletes’ ankle sprains are managed conservatively with excellent outcomes and full return to their pre-injury level of play. However, it is essential to differentiate the single ligament sprain from a more complex injury to the ankle joint. The evidence-based treatment and rehabilitation programmes are associated with a better prognosis and a faster time to return to sport participation.

## Introduction

Contact sports are associated with high overall injury rates, both at the professional and amateur level. End-stage ranges in mobility, speed, and directional changes are known to increase the number of injuries [1]. For the ankle, the most common condition in this regard is the so-called “ankle sprain.”

The incidence of ankle sprains has been reported to be between 0.324 and 9 per 1000 h of activity [1, 2]. This variability can be related to differences in injury nomenclature and non-homogenous studied populations. Higher ankle sprain rates have been reported predominantly in older athletes, dominant leg, during official games and occurring at the end of each half of a football match [3]. Approximately 60% of all ankle sprains in athletes arise as a result of direct trauma or due to contact [4, 5]. The overall ankle re-sprain rate in contact sports is reported to be between 4 and 29% [3–5].

The ankle sprain used to be the most common injury type in professional football players representing 10% to 36% of all injuries [6, 7], but recent studies proclaim a lower ankle injury rate, representing 10 to 15% of all injuries [7, 8]. Potential reasons for this declining trend involve successful injury prevention strategies (e.g. balance training and bracing/taping), stricter game rules, and a more detailed reporting culture of specific injury subtypes [5].

Ankle injuries are nowadays the fourth most common injury type in elite football, and they are preceded by the knee, the thigh, and the lower leg [7, 8]. Moreover, a recent long-term ankle injury study documented an injury rate of 1/1000 h [5]. This means practically that a professional 25-player team can expect approximately 7 ankle injuries per season. In terms of overall mean time loss, this represents an average of 16–24 calendar days for every ankle sprain [1, 3, 5, 9]. In case of a more severe ankle sprain, this mean number rises to approximately 28 days of absence. In elite football, the ankle sprain represents 10 to 17% of all related ankle injuries [5, 8, 9].

## Joint Anatomy

The ankle joint can be mechanically seen as a fork, in which the tibia and both malleoli form a mortise to accommodate the talar bone. As a hinge joint, there is a single axis of movement that allows for dorsal flexion (50°) and plantar flexion (20°). The talar bone has a narrower superior surface posteriorly that leads to a looser fit when the fork is moving into plantar flexion. The function of the ankle ligaments is to provide the ankle with the necessary postural stability. The reduced intrinsic intra-articular stability during plantar flexion could explain why most ligamentous injuries are seen in plantar flexion [10]. Inferior to the talocrural joint, the subtalar joint is formed between the inferior surface of the talus and the superior surface of the calcaneus. This subtalar joint provides 35° of inversion and 15° of eversion.

**Fig. 1:**
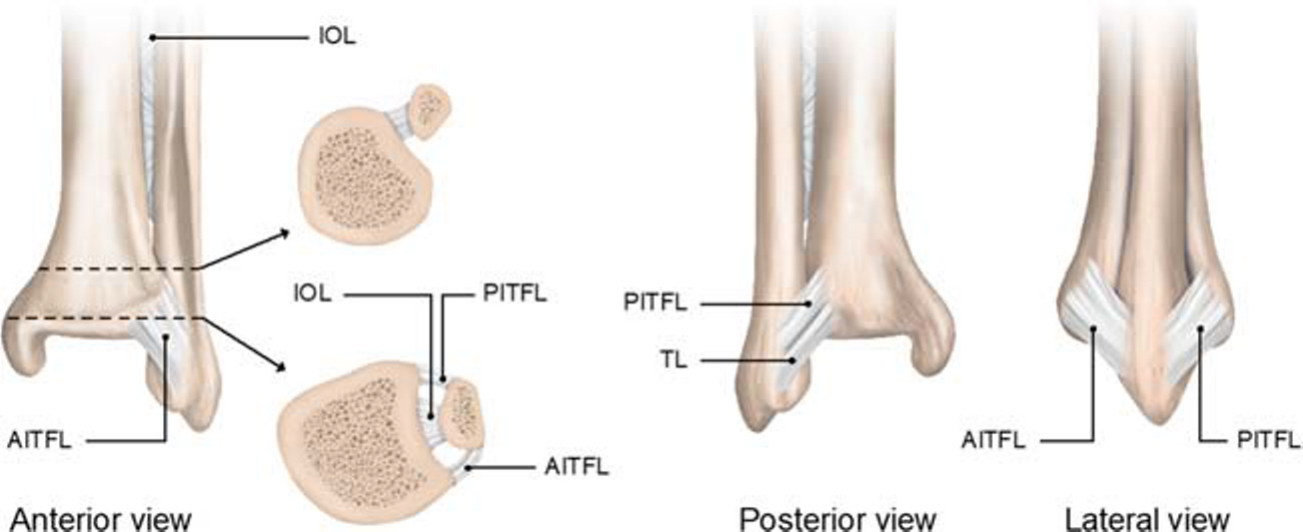
The syndesmotic ankle ligaments in 3 views presented

The fibula and tibia also articulate distally, creating an inferior tibiofibular joint (supported by the syndesmotic ligaments), and they form the inferior or distal tibiofibular joint (named as “distal tibiofibular syndesmosis”—Fig. 1). It is the syndesmotic joint that allows the tibia-fibula complex to adapt as a whole to the varying width of the upper articular surface of the talar bone, and this minimal movement is vital for enabling normal walking and running. Both passive and dynamic factors provide stability to the ankle joint. Passive stabilization relies on the morphology of the articular surfaces, the articular capsule, surrounding ligamentous complexes, and the retinacula. Dynamic balance is provided by muscle activation mainly.

## Lateral Ligament Complex Injury

Ankle sprains account for 67 to 72% of all football player-related injuries to the ankle [3, 5, 9, 11, 12]. The ankle joint sprain rate in amateur and professional football players is 2.16 and 0.17 per 1000 h of exposure, respectively [5, 11]. Most ankle injuries in footballers are caused by player’s contact, direct trauma, mostly during tackling (54%) [9]. Foul play is involved in 40% of the match-related ankle injuries [5].

Previous sprain injury at the ankle increases 2 to 5 times the chance to have a recurrent ankle sprain compared with players without previous injury [11, 13, 14]. There are no significant gender differences in the overall incidence rate of ankle sprains [15, 16]. A mean lay off per ankle sprain in football is reported between 7 and 18 days [5, 9, 11]. A total of 83–89% of the ankle sprains require athletes less than 4 weeks of loss of activities [5, 8, 9], suggesting that it is the incidence rather than the severity of ankle sprains that makes them problematic [9].

However, after standard treatment for an acute sprain ankle, up to 40% of the patients in the general population report residual symptoms [17, 18]. These most common residual symptoms are chronic pain, recurrent instability, and muscular weakness. The reported mean costs per ankle sprain are €360.60 ± 426.73 [19], but these are unmistakably higher in elite sports.

## Aetiology

Injury to the lateral ligamentous complex represents 70–91% of all ankle sprains in elite football players [9, 11, 20, 21]. This can be partially explained by the relative weakness of the lateral ligaments and the natural tendency for the ankle to go into inversion. The most common mechanism of injury is the inversion of the plantar-flexed foot.

Repetitive video analysis of ankle sprains in football revealed two common mechanisms that put the ankle in this vulnerable position [1]: impact by an opponent on the medial aspect of the lower leg just before or at foot strike, resulting in a laterally directed force causing the footballer to land with the ankle in a vulnerable inverted position [2]. Forced plantar flexion when the injured footballer hits the foot of the opponent when attempting to shoot on the goal or clear the ball [22].

**Fig. 2:**
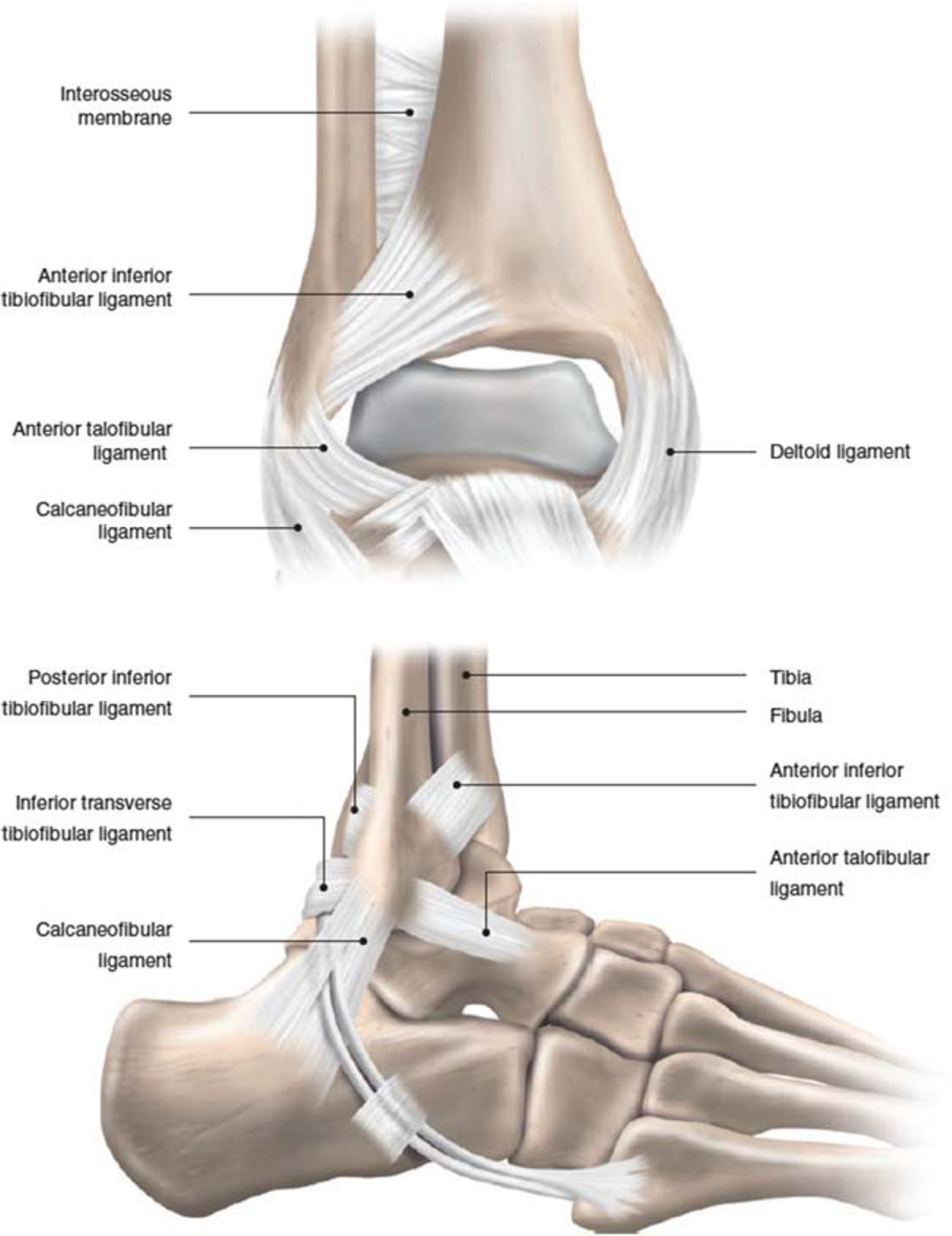
Antero-posterior and lateral view to the lateral ankle joint ligaments

As the anterior talo-fibular ligament (ATFL) is maximally stretched during inversion of the plantar-flexed foot and as it has the lowest tolerance to loads (approximately 150 N [23, 24]), the ATFL is the first and often the only ligament injured. As a result, the ATFL is the most frequently injured ligament of the ankle (90–95% [9, 11]). When the mechanism of injury continues around the lateral aspect of the ankle, rupture of the Anterior Talo-Fibular Ligament (ATFL) can cascade to the Calcaneo-Fibular Ligament (CFL), and finally (less frequently) to the PTFL (Fig. 2). It was reported in an MRI study that 41% of the patients with an ankle inversion trauma have injured both the ATFL and CFL, whereas only 5% had damaged the PTFL [25]. Associated injuries include bony fractures, osteochondral lesions, and damage to both the peroneus tendon and nerve.

## Clinical Findings

Patient history taking and video analysis are found very useful in studying the injured ligaments. It is essential to distinguish a simple distortion from a lateral ligament rupture since adequate treatment is associated with a better prognosis and time to return to play [26, 27]. Athletes together with recreants typically report a sudden twisting of the ankle joint, smaller ability to bear weight, and they usually can identify the palpatory painful spot. Patients with lateral ligamentous ruptures report more immediate joint oedema and are more frequently prone to stop their activities [28]. An audible snap or crack sound may accompany ligament sprains. Bony structures and all ligamentous entities around the ankle joint should be palpated for tenderness, including the fibular bone and the base of the fifth metatarsal on the lateral side of the foot. If there is no pain upon palpation over the ATFL, there is probably no lateral ligament rupture apparent [26, 27].

Note that approximately 40% of the athletes with a lateral ligament rupture present with pain upon palpation over the medial malleolus, whereas 60% report tenderness upon palpation over the AITFL (without rupture of this ligament), probably due to a tear of the anterior capsule [28]. For the physical examination, the anterior drawer test evaluates the ATFL instability, whereas the talar tilt test aims at identifying CFL (calcaneus fibular ligament) instability. If an ecchymosis is present, accompanied by palpatory pain or a positive stress test (or both), it is most likely that a partial lateral ligamentous rupture exists [26, 27].

However, especially in the acute phase, manual stress tests are less reliable due to inhibiting swelling and pain. Therefore, delayed physical examination (after 4–5 days post-injury) of the ankle joint is more reliable and therefore considered the golden standard for diagnosing acute injury to the lateral ligament complex of the ankle. Diagnosing an acute lateral ligament rupture during a delayed physical examination has a sensitivity of 96%, with a specificity of 84% [26, 27].

## Diagnosis

The Ottawa ankle rules help to determine if X-rays are indicated in the assessment of an acute ankle sprain. These Ottawa rules are an instrument to rule out suspected fractures over the ankle after ankle sprain and have a sensitivity close to 100%. Stress radiographs often are not indicated in the routine diagnosis of lateral ligament sprain, as they are challenging to perform and have a relatively low impact on the treatment protocol.

Ultrasound (US) and magnetic resonance scan (MRI) can be useful in the diagnosis of associated injuries (bone, chondral, or tendon). The US has demonstrated to be an accurate tool for investigation of ligament sprains but may be difficult to interpret on retrospective review by other physicians. The sensitivity and specificity of US for a lateral ligament injury is 92% and 64%, respectively [27]. When US is performed after an inconclusive delayed physical examination, the sensitivity increases to 100% and specificity to 72% [27]. A recent study of ATFL injury comparing ultrasonography in the emergency room with MRI images found no overall differences in diagnostic accuracy [29].

**Fig. 3:**
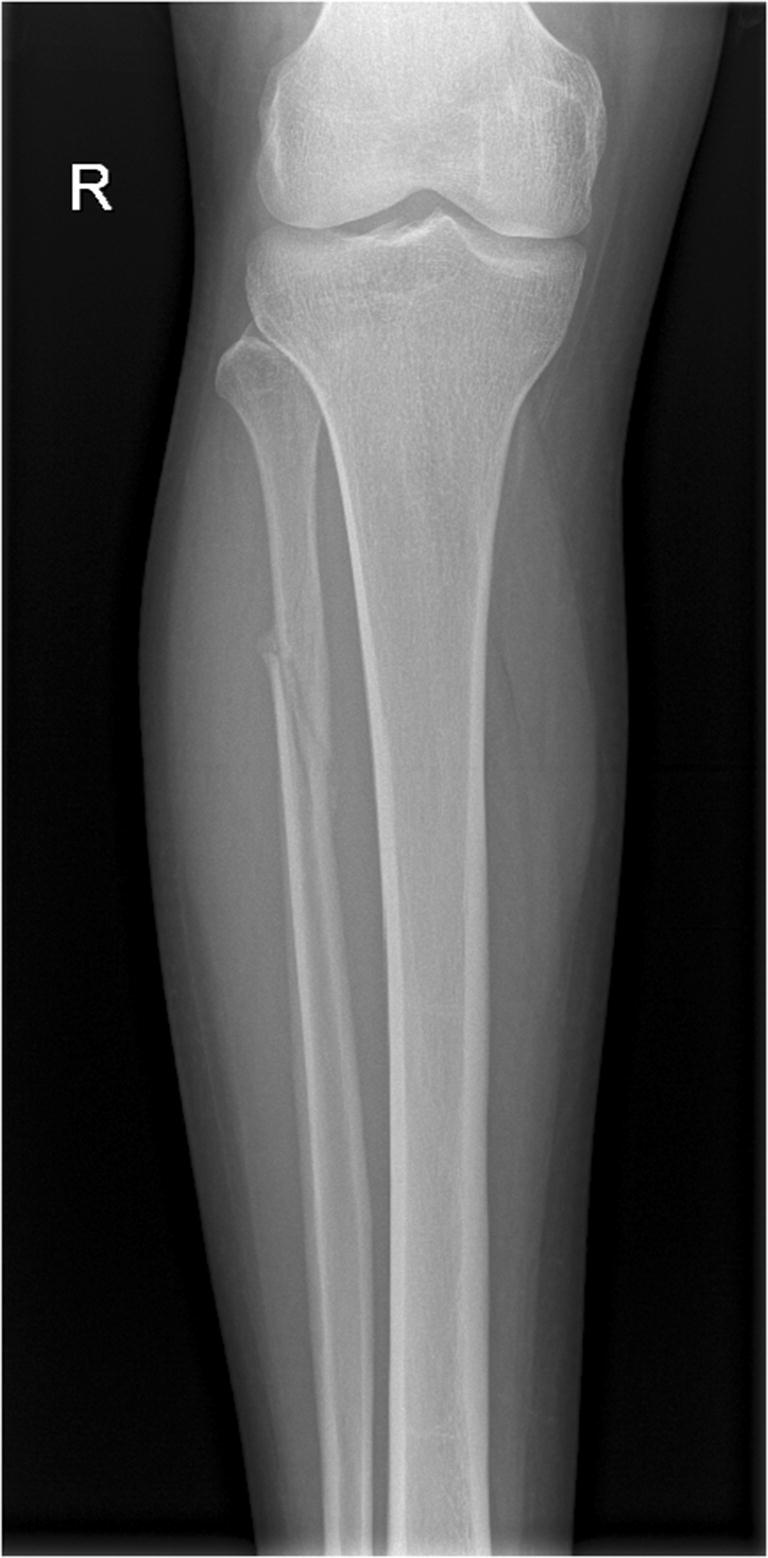
Maisonneuve fracture after an external rotational injury to the ankle

MRI is a reliable tool in the diagnosis of injury to the lateral ligamentous ankle complex and associated injuries (Fig. 3), including tendinous and syndesmotic trauma, osteochondral lesions, and occult fractures. The sensitivity and specificity of MRI for ATFL injuries are 92–100% and 100%, respectively [30, 31]. In comparison with arthroscopy, MRI images correctly located the injured portion of the ATFL in 93%, whereas US was able to identify in 63% [31]. Overall, MRI is the imaging modality of choice for lateral ligamentous injuries over the ankle in elite sports.

## Treatment

A grading system has been created to aid in guiding the treatment of lateral ligament injuries [32, 33]. This system incorporates anatomical injury with clinical symptoms and is only reliable with delayed physical assessment.

Grade I (mild) injuries include ligament fibre stretch without macroscopic rupture. There is clinically minor swelling and palpatory tenderness, hardly any functional loss, and no increased instability.

Grade II (moderate) injuries include partial ligament tear with moderate pain, swelling and palpatory tenderness, mild to moderate instability, and moderate functional disability.

In grade III (severe) injuries, a complete tear of the ligament and joint capsule rupture is present combined with severe bruising, swelling, and pain. There is a significant loss of function and an increased instability. The athlete is unable to bear weight and walk normal. In clinical treatment practice, only the difference between a simple sprain (grade I) and real instability (grade III) is relevant.

Although there exists a substantial body of research on ankle injuries, controversy remains concerning the best treatment for acute lateral ligament injuries, especially for grade III injuries in elite athletes. However, non-operative measures can be prescribed for the majority of acute grades I–III lateral ligament sprains with good to excellent outcome [34].

Treatment of lateral ligament tears is based on the 3 phases of biological ligament healing: inflammatory phase, proliferation phase (6 to 12 weeks post-trauma), and remodelling or maturation phase (until 1-year post-injury).

Initially, the treatment during the inflammatory phase is aimed towards avoiding swelling and ongoing injury. This is achieved with the POLICE (protect, optimal loading, ice, compression, elevation) protocol for the first 4–5 days [35]. Oral or topical use of nonsteroidal anti-inflammatory drugs (NSAID) decreases pain without increase in adverse events [36]. Manual mobilization therapy of the ankle joint has minimal benefits and should be discouraged [37]. No effect was found from therapies like low-intensity ultrasound, laser, or electrotherapy [37]. Long-term immobilization (> 2 weeks) leads to poorer outcomes than functional treatment [34, 38]. However, a short time of immobilization (maximum 10 days) in a cast below the knee or removable boot can be advantageous for severe lateral ligamentous tears (faster recovery compared with compression bandage) [34, 39].

In the proliferation phase, fibroblasts proliferate and new collagen is formed. Controlled stress on the damaged ligament will promote proper collagen fibres orientation, whereas protection of ankle inversion is essential to prevent the excess formation of weaker type III collagen.

Hereby, the prescription of an external ankle brace is advocated. Treatment with elastic bandages results in fewer complications versus taping but is associated with a slower return to sport and remaining instability versus a semi-rigid ankle brace [40]. Although a recent study reported no differences in outcome 6 months after treatment with tape, semi-rigid brace, or lace-up brace [41], a significant number of studies report superior results from protection with a brace [34, 42]. The use of a (semi-rigid) brace in the proliferation phase is preferred since it is more cost-effective and leads to fewer skin complications. Exercise therapy combined with progressive weight bearing is also an essential stage in the functional treatment of acute lateral ligamentous sprain [43]. Early active range of motion (ROM) exercises are subsequently followed by strengthening exercises, proprioceptive training, and functional exercises. Activities in this final phase should progressively simulate the physical demands of the respective sport modality during practise sessions and competition that includes jumping, turning, and twisting. Supervision by a multidisciplinary team and knowledgeable sport medicine staff is necessary and can be considered for all athletes, although unsupervised, home-based training was also reported to be effective [44]. Ideal rehabilitation programme schedules for acute lateral ligamentous injuries, based on current best evidence, have been described [45–47]. However, there is still a lack of research to design specific rehabilitation protocols for returning athletes to sports competition.

The treatment of grade III lateral ligament ankle injury remains controversial. Most reviews comparing surgery versus conservative treatment for acute lateral ankle ligament injuries failed to demonstrate a superior treatment approach [34, 42]. Therefore, functional treatment is preferred over surgery in most of the cases [34, 42]. However, surgical treatment may be beneficial on an individual basis in elite athletes [48]. The advantage of surgical repair is significantly less objective instability when compared with non-operative treatment [43]. Since increased instability is predictive for future ankle sprains [49] and return to sports is not delayed after surgical treatment [48], acute surgical repair should be considered in professional football players [28, 34, 48].

Timing in the season, expectations of the athlete, sports-specific ankle load, individual history, stage of athlete career, time from trauma to diagnosis, combined injury, and access to expert medical imaging and treatment are all features to be assessed when considering surgery [28]. When indicating a surgical repair in an acute injury, a direct anatomical reconstruction of the ruptured lateral ligaments in a high-volume centre by an experienced ankle sports surgeon is recommended [28, 48]. The rehabilitation regime after direct anatomic reconstruction, as described in a recent evidence-based guideline [50], is lower-leg cast for 1 or 2 weeks, followed by 2–4 weeks in a walking boot and an active rehabilitation protocol with the use of an ankle support.

## Return to Play

After a lateral ligamentous injury, it is difficult to predict precisely when the athlete can return to sports (RTS). Furthermore, residual disability of ankle joint sprains is often caused by an inadequate rehabilitation programme and early RTS [46]. The current literature lacks formal criteria to assist in the decision to RTS of athletes with a lateral ligament injury. When analyzing the ability of an athlete to return to sports activities, all functional limitations as a result of the damage have to be restored, cardiovascular fitness should be equal to or greater than pre-injury status, and there should be no apprehension from the athlete or other members of the rehabilitation team concerning the health safety of the athlete. The RTS process itself will often be progressive as well, and objective data are required to assess the ability of the athlete to progress to the next rehabilitation phase. Although self-reported ankle scoring systems (e.g. FAOS [51]) are not validated for RTS decisions, they can be useful to evaluate the effectiveness of the rehabilitation protocol. Moreover, the use of functional performance tests is considered helpful to assess the ability to perform sport-specific athletic skills again [45].

Tests can progress from relatively simple tasks (like the single-legged balance test [52]) to more complex tasks (such as the Star Excursion Balance Test [53], the Y-balance test [54], and the agility T-test [55]). The outcome of these tests should be evaluated throughout the rehabilitation process, thereby quantifying progress and comparison against pre-injury level and the contralateral side. As several functional tests are predictive of ankle injuries in uninjured athletes [55–58], the use of these tests in the RTS decision of athletes with lateral ligamentous ankle injury should be validated. A minimal score on the functional test for RTS, e.g. 90% of pre-injury or contralateral side, has been advised [44] but warrants further research [45]. In the case of RTS, the rehabilitation programme should never be stopped abruptly, as deficits can erroneously be overlooked during the return to play evaluation. Moreover, specific gaps may only be present after the athlete has been thoroughly fatigued. Continuing sport-specific rehabilitation will help to minimize this risk.

The time needed to RTS in lateral ligamentous ankle sprains depends on several factors, including the severity of the injury, the ability of the athlete, and the rehabilitation features available. The reported RTS in amateur and professional football players has been between 7 and 15 ± 19 days, respectively [5, 11]. There was no documentation on the gradation of the injuries. A case series of professional athletes who underwent surgical ligament repair reported a median RTS of 77 days for isolated lateral ligamentous injuries and 105 days for those with concomitant injuries [50].

The most critical risk factor for an ankle sprain is the history of a previous ankle sprain due to a reduced mechanical stability and reduced proprioceptive ability. There is evidence that neuromuscular training, especially balance training (e.g. wobble board), is useful in the prevention of recurrent ankle sprains. This type of therapy can also be effectively performed at home [44]. It is controversial if neuromuscular training is beneficial in healthy ankles in preventing the first presentation of a sprain [28]. There is a consensus that external ankle brace reduces the risk of recurrent ankle injury in previously injured athletes [40] by approximately 70% [59]. These results were reproduced in football players [7, 60]. It is unclear whether an external brace is more effective than taping [48], since both have their advantages and disadvantages. The taping technique can lead to skin lesions and loses 40–50% of its effectiveness after 15 min of intensified exercise [61]. However, some athletes tend to dislike braces because they do not fit well in the usual football shoes. Braces are re-usable and re-adjustable, and minimal expertise is required for correct installation. Contrary to popular belief, external ankle support does not impede speed, agility, and kicking accuracy in football players [62, 63]. A combination of both treatment modalities can therefore be considered.

## Fact Box

- Delayed physical examination (4–5 days) of the lateral ankle ligament complex gives better results than the one that is done within 48 h.
- The majority of acute lateral ligament injuries of the ankle can be treated conservatively with an adequate rehabilitation protocol.
- Surgical treatment can be considered in high-level athletes with acute grade III injuries.
- Surgery provides a lower incidence of chronic ankle instability versus conservative treatment.
- RTS criteria should include functional performance tests (e.g. 90% score)

## Conclusions

This review describes the current evidence on the most common sports-related ankle injuries with a specific focus on the available evidence towards return to play. The majority of the athletes’ ankle sprains are managed conservatively with excellent outcomes and full return to their pre-injury level of play. More severe—grade III—lateral ligament ankle injury remains controversial. Acute surgical repair should be considered in professional football players, especially when increased instability is present. RTP management is a multifactorial and interdisciplinary process. Individualized assessment should focus on patient profile, injury type, and sports modality.
